# Beyond BMI: Personalized Nutrition in Obesity, Normal-Weight Obesity, Metabolic Syndrome, and MASLD

**DOI:** 10.3390/nu18142345

**Published:** 2026-07-17

**Authors:** Aldona Wierzbicka-Rucińska

**Affiliations:** Department of Clinical Biochemistry, The Children’s Memorial Health Institute, Aleja Dzieci Polskich 20, 04-730 Warsaw, Poland; a.wierzbicka-rucinska@ipczd.pl

**Keywords:** personalized nutrition, precision nutrition, obesity, normal-weight obesity, metabolic syndrome, metabolic dysfunction-associated steatotic liver disease, nutrigenetics, gut microbiota, metabolic phenotyping, body composition, digital health

## Abstract

**Background:** Personalized nutrition, also referred to as precision nutrition, is an emerging approach that integrates genetic, metabolic, phenotypic, behavioral, and environmental characteristics to develop individualized dietary strategies. Obesity, metabolic syndrome (MetS), and metabolic dysfunction-associated steatotic liver disease (MASLD) represent interconnected disorders with substantial inter-individual variability in disease development, metabolic risk, and response to dietary interventions. Although body mass index (BMI) remains widely used for obesity classification, it does not adequately capture differences in body composition, fat distribution, or metabolic health. Consequently, individuals with normal-weight obesity (NWO), characterized by excessive body fat accumulation despite a normal BMI, may remain unidentified despite increased cardiometabolic risk.This narrative review critically evaluates the current evidence on the potential role of personalized nutrition in the prevention and management of obesity, MetS, MASLD, and related cardiometabolic abnormalities. Particular attention is given to five major domains: nutrigenetics, gut microbiota, metabolic phenotyping, body composition assessment, and digital health technologies, with emphasis on their current clinical applicability and limitations. **Methods:** A structured narrative review was performed using PubMed, Scopus, and Web of Science to identify English-language studies (2003–2026) on personalized nutrition in obesity, normal-weight obesity, metabolic syndrome, and MASLD. Eligible studies were selected according to predefined inclusion and exclusion criteria, and 31 publications were included in the qualitative synthesis. **Results:** Current evidence suggests that personalized nutrition strategies may contribute to improvements in body weight regulation, insulin sensitivity, lipid metabolism, and liver-related outcomes; however, the magnitude and consistency of these effects remain variable. The integration of genetic, metabolic, microbiome, and phenotypic information may improve individual risk stratification and help identify high-risk groups, including individuals with NWO who may not be recognized through BMI-based assessment alone. Emerging approaches involving multi-omics technologies, microbiome profiling, wearable devices, continuous glucose monitoring, and artificial intelligence-based tools provide promising opportunities for individualized dietary interventions. Nevertheless, limitations related to methodological heterogeneity, insufficient standardization, limited external validation, and the scarcity of long-term pragmatic clinical trials currently restrict their routine implementation. **Conclusions:** Personalized nutrition represents a promising but still evolving approach for addressing obesity and its metabolic complications, including MetS and MASLD. While the integration of biological, phenotypic, and digital information may support more targeted dietary recommendations, current evidence does not yet fully establish the clinical effectiveness and cost-effectiveness of these approaches in routine care. Future large-scale, longitudinal, and well-designed randomized controlled trials are required to determine which personalized nutrition strategies provide clinically meaningful benefits and for which patient populations.

## 1. Introduction

Obesity and its associated metabolic complications represent one of the most significant public health challenges of the twenty-first century. The global prevalence of obesity continues to increase across all age groups, contributing substantially to the growing burden of type 2 diabetes mellitus, cardiovascular disease, metabolic dysfunction-associated steatotic liver disease (MASLD), impaired quality of life, and premature mortality [1,2,3,4,5]. In addition to its clinical consequences, obesity generates considerable socioeconomic costs through increased healthcare utilization, disability, and productivity losses [2,3]. Among obesity-related metabolic disorders, metabolic syndrome (MetS) and MASLD are of particular importance because they share overlapping pathophysiological mechanisms, including insulin resistance, chronic low-grade inflammation, dysregulated lipid metabolism, and altered energy homeostasis. The frequent coexistence of these conditions substantially increases the risk of cardiovascular disease and other metabolic complications. For decades, body mass index (BMI) has been the primary tool for defining obesity because of its simplicity, low cost, and applicability in clinical practice and epidemiological studies [6]. However, BMI provides limited information regarding body composition, fat distribution, and metabolic health, as it does not distinguish between adipose and lean tissue or adequately reflect visceral adiposity [4,5,6,7]. Consequently, individuals with identical BMI values may exhibit markedly different cardiometabolic risk profiles. A clinically relevant example of this limitation is normal-weight obesity (NWO), a phenotype characterized by excessive body fat accumulation despite a BMI within the normal range. Individuals with NWO may present insulin resistance, dyslipidemia, chronic inflammation, metabolic syndrome, and increased cardiovascular risk despite being classified as having normal body weight according to conventional BMI criteria [4,6,8,9,10,11,12,13,14,15,16,17,18]. These observations highlight the need for a more comprehensive assessment of body composition and metabolic function beyond BMI-based classification. Additional measures, including waist circumference (WC), waist-to-hip ratio (WHR), and advanced body composition techniques, may provide clinically relevant information regarding abdominal adiposity and metabolic risk. Lifestyle modification, including dietary intervention and increased physical activity, remains the foundation of obesity, MetS, and MASLD management. However, accumulating evidence indicates substantial inter-individual variability in response to similar dietary approaches. Differences in postprandial glucose regulation, lipid metabolism, weight loss outcomes, and metabolic adaptations have been associated with variations in genetic background, gut microbiota composition, metabolic phenotype, behavioral characteristics, and environmental exposures [19,20,21,22,23]. These findings have challenged the traditional population-based “one-size-fits-all” dietary approach and contributed to the emergence of personalized nutrition as an important component of precision medicine. Personalized nutrition, also referred to as precision nutrition, integrates multiple sources of individual-level information, including genetic, metabolic, phenotypic, behavioral, and environmental characteristics, to develop more targeted dietary recommendations [19,20,21,22,23,24]. Potential applications include improved identification of individuals at increased metabolic risk, optimization of dietary interventions, and more effective prevention and management of obesity-related complications. Nevertheless, despite rapid advances in this field, the clinical implementation of personalized nutrition remains limited. Current evidence is heterogeneous, and uncertainties persist regarding the predictive value of genetic markers, reproducibility of microbiome-based approaches, standardization of metabolic phenotyping, accessibility of advanced body composition assessment, and the real-world applicability of digital health technologies. Although numerous reviews have examined individual components of precision nutrition, such as nutrigenetics, gut microbiota, metabolomics, or digital health applications, relatively few have evaluated these approaches within an integrated framework encompassing obesity, normal-weight obesity, metabolic syndrome, and MASLD. Moreover, the extent to which these complementary domains can be combined to support clinically meaningful and individualized nutritional strategies remains insufficiently defined. Therefore, the aim of this narrative review is to critically evaluate current evidence regarding the role of personalized nutrition in the prevention and management of obesity, NWO, MetS, and MASLD. By integrating findings from five complementary domains—nutrigenetics, gut microbiota, metabolic phenotyping, body composition assessment, and emerging digital health technologies—this review seeks to provide a comprehensive perspective on current opportunities, limitations, and future directions for translating precision nutrition into clinical practice.

## 2. Materials and Methods

### 2.1. Study Design

This narrative review was conducted using a structured literature search strategy to provide a comprehensive and critical overview of current evidence regarding personalized nutrition in obesity, normal-weight obesity (NWO), metabolic syndrome (MetS), and metabolic dysfunction-associated steatotic liver disease (MASLD). The review aimed to integrate clinical, translational, and mechanistic evidence from different research domains, while considering the methodological limitations and varying levels of evidence provided by different study designs. Relevant publications were identified according to predefined eligibility criteria and narratively synthesized. Given the broad and multidisciplinary nature of personalized nutrition research, including intervention studies, observational analyses, mechanistic investigations, and emerging technology-based approaches, a qualitative synthesis was considered the most appropriate methodological approach.

### 2.2. Literature Search Strategy

A structured literature search was performed in PubMed, Scopus, and Web of Science databases to identify relevant publications published between January 2003 and March 2026. These databases were selected to capture biomedical, clinical, and interdisciplinary research related to nutrition science, metabolic disorders, and precision medicine. The search strategy combined controlled vocabulary and free-text terms related to personalized nutrition and obesity-associated metabolic disorders. The following search terms were used in various combinations: (“personalized nutrition” OR “precision nutrition” OR “individualized nutrition”) AND (obesity OR “normal-weight obesity” OR “metabolic syndrome” OR MASLD OR “metabolic dysfunction-associated steatotic liver disease”) AND (nutrigenetics OR nutrigenomics OR microbiome OR “gut microbiota” OR metabolomics OR phenotyping OR “body composition” OR “digital health”). Additional relevant publications were identified through manual screening of reference lists from included studies and recent reviews. Only studies published in English and involving human participants were considered eligible.

### 2.3. Eligibility Criteria

Studies were included if they met the following criteria: investigated personalized or precision nutrition approaches in obesity, NWO, MetS, or MASLD; evaluated dietary interventions, nutrigenetics, nutrigenomics, gut microbiota-based approaches, metabolomics, metabolic phenotyping, body composition assessment, or digital health technologies; included randomized controlled trials, prospective cohort studies, observational studies, systematic reviews, meta-analyses, translational studies, or relevant consensus documents. Studies were excluded if they were conference abstracts without full-text availability; were editorials, commentaries, or letters; involved animal or in vitro models unless findings were considered essential for mechanistic interpretation; or did not directly address personalized or precision nutrition.

### 2.4. Study Selection

**Figure 1:**
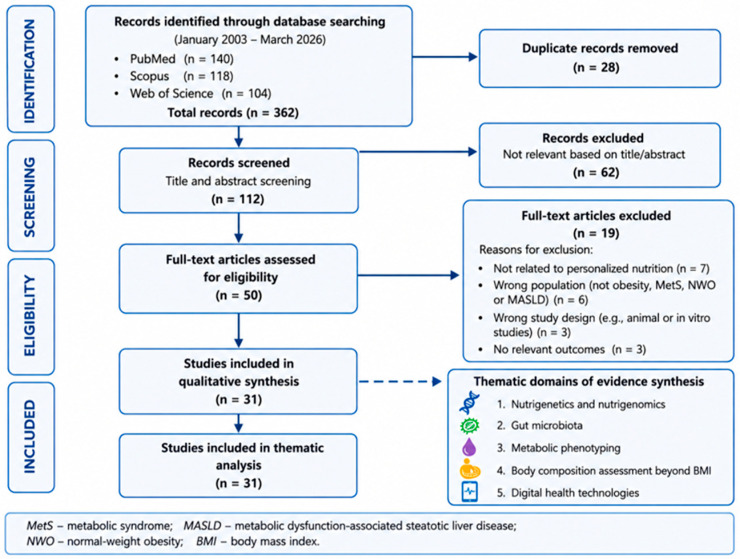
Methodological workflow of the narrative review. The review included a PubMed search (January 2003–March 2026), screening and selection of relevant studies on personalized nutrition in obesity, metabolic syndrome (MetS), and metabolic dysfunction-associated steatotic liver disease (MASLD), followed by data extraction, publication trend analysis, and qualitative evidence synthesis.

Titles and abstracts retrieved from the databases were screened according to predefined eligibility criteria. Articles considered potentially relevant underwent full-text assessment. The initial search identified 140 records. After removal of duplicates, 112 records remained for title and abstract screening. Of these, 62 articles were excluded because they did not fulfill the inclusion criteria. A total of 50 full-text articles were assessed for eligibility, and 19 publications were excluded because of lack of relevance to personalized nutrition, inappropriate study design, non-human populations, or insufficient outcome information. Ultimately, 31 studies were included in the qualitative narrative synthesis. The study selection process is presented in Figure 1. The flow diagram is provided to improve transparency of study identification and selection; however, this review was not designed as a systematic review or meta-analysis.

### 2.5. Data Extraction

For each included publication, the following information was extracted: study design, study population characteristics, metabolic condition investigated, personalized nutrition approach, intervention characteristics, primary outcomes, main findings, and potential clinical implications. Data extraction was performed to facilitate comparison across research domains and to identify common themes, areas of uncertainty, and knowledge gaps relevant to personalized nutrition.

### 2.6. Evidence Synthesis

Due to substantial heterogeneity in study designs, populations, interventions, outcomes, and methodological approaches, quantitative meta-analysis was not performed. Instead, findings were synthesized narratively. The interpretation of evidence considered the methodological characteristics and relative strengths of different study designs. Randomized controlled trials were considered the primary source of evidence for evaluating intervention effectiveness, whereas observational studies contributed information regarding associations and real-world applicability. Mechanistic studies, including investigations of genetics, microbiota, metabolomics, and biological pathways, were used to provide insight into potential mechanisms but were not interpreted as direct evidence of clinical effectiveness. Evidence was organized into five thematic domains representing major components of personalized nutrition:

Nutrigenetics and nutrigenomics;

Gut microbiota;

Metabolic phenotyping;

Body composition assessment beyond BMI;

Emerging digital health technologies.

This framework enabled an integrated evaluation of personalized nutrition approaches across obesity, NWO, MetS, and MASLD, while emphasizing current limitations, translational challenges, and priorities for future research.

Because personalized nutrition research includes heterogeneous study designs, evidence from randomized controlled trials was interpreted primarily for clinical efficacy, whereas cohort, mechanistic, and multi-omics studies were considered supportive evidence regarding biological variability and potential predictors of response.

## 3. Results

### 3.1. Overview of the Included Evidence

The structured literature search identified studies evaluating personalized nutrition across obesity, normal-weight obesity (NWO), metabolic syndrome (MetS), and metabolic dysfunction-associated steatotic liver disease (MASLD). The final evidence base included randomized controlled trials, prospective cohort studies, observational studies, systematic reviews, meta-analyses, and translational investigations. Most studies were conducted in adult populations with overweight or obesity, whereas fewer focused specifically on NWO or MASLD. Considerable heterogeneity was observed in study design, population characteristics, intervention strategies, follow-up duration, and outcome measures. Despite this variability, five core domains consistently emerged as key components of personalized nutrition: nutrigenetics, gut microbiota, metabolic phenotyping, body composition assessment, and digital health technologies.

An overview of the representative studies included in this review, together with their study designs, personalization strategies, and principal findings, is presented in Table 1.

### 3.2. Nutrigenetics and Nutrigenomics

Nutrigenetics represents one of the most extensively studied components of personalized nutrition. Evidence indicates that genetic variability influences appetite regulation, energy balance, lipid metabolism, insulin sensitivity, and individual responses to dietary interventions. Polymorphisms in genes such as FTO, MC4R, PPARG, and TCF7L2 have been associated with differences in weight-loss response and cardiometabolic outcomes. However, effect sizes are generally modest and inconsistent across populations, limiting the predictive utility of genetic information when used in isolation. In obesity and MetS, integrating genetic data with dietary counseling may slightly improve adherence and behavioral outcomes, although consistent long-term benefits on weight reduction or metabolic improvement remain unproven. Evidence in MASLD is still emerging and largely observational, suggesting that genetic susceptibility may modulate hepatic fat accumulation and dietary responsiveness. Overall, current data support nutrigenetics as a complementary tool rather than a standalone predictor in precision nutrition.

### 3.3. Gut Microbiota as a Determinant of Dietary Response

The gut microbiota plays a central role in inter-individual variability in metabolic responses to diet. Microbial composition, diversity, and metabolite production have been associated with obesity, insulin resistance, systemic inflammation, and hepatic steatosis. Landmark precision nutrition studies have shown that microbiome profiles improve the prediction of postprandial glycemic responses beyond traditional clinical markers. These findings support the concept that microbial signatures can help identify individuals who may benefit from specific dietary patterns. In obesity and MetS, microbiome-targeted interventions have been associated with improvements in glycemic control, lipid metabolism, and inflammatory markers. In MASLD, dysbiosis, altered gut–liver axis signaling, and increased intestinal permeability are increasingly recognized as key contributors to disease progression. Despite promising results, translation into clinical practice remains limited by methodological variability, lack of standardized analytical pipelines, and insufficient validation in large randomized trials.

### 3.4. Metabolic Phenotyping

Metabolic phenotyping provides a functional approach to stratifying individuals based on metabolic responses rather than anthropometric measures alone. Evidence shows that insulin sensitivity, postprandial glycemic responses, lipid metabolism, resting energy expenditure, and metabolomic profiles strongly influence dietary responsiveness. Individuals with similar clinical characteristics often exhibit markedly different metabolic responses, highlighting substantial metabolic heterogeneity. Large-scale studies, including the PREDICT cohorts, have demonstrated that combining metabolic biomarkers with clinical and lifestyle data significantly improves the prediction of individual dietary responses. In obesity, MetS, and early MASLD, metabolic phenotyping may help identify individuals more likely to respond to specific dietary strategies such as Mediterranean, low-carbohydrate, or energy-restricted diets. Many of these biomarkers are already available in routine clinical practice, making this approach one of the most clinically translatable components of precision nutrition.

### 3.5. Body Composition Assessment Beyond BMI

Across the reviewed literature, a consistent limitation of BMI is its inability to capture body fat distribution and metabolic risk. Individuals with normal-weight obesity may present increased visceral adiposity, insulin resistance, chronic inflammation, and elevated cardiometabolic risk despite normal BMI values. Conversely, some individuals classified as obese by BMI may maintain relatively preserved metabolic health. Body composition assessment methods—including dual-energy X-ray absorptiometry (DXA), bioelectrical impedance analysis (BIA), waist circumference, waist-to-height ratio, and imaging-based evaluation of ectopic fat—provide substantially more precise information on metabolic risk than BMI alone. In obesity, MetS, and MASLD, abdominal fat distribution and ectopic fat deposition show stronger associations with insulin resistance and hepatic steatosis than total body weight. These findings support the integration of body composition assessment into routine clinical evaluation to improve risk stratification and personalize nutritional interventions.

### 3.6. Emerging Digital Health Technologies

Digital health technologies have significantly expanded the feasibility of personalized nutrition. Tools such as continuous glucose monitoring, wearable activity trackers, mobile health applications, artificial intelligence, and machine learning algorithms enable real-time assessment of dietary intake, physical activity, sleep patterns, and metabolic responses. When integrated with multi-omics data (genomics, metabolomics, and microbiome profiling), these technologies facilitate the development of predictive models capable of generating individualized dietary recommendations. Evidence suggests that digital health interventions may improve dietary adherence, patient engagement, and long-term lifestyle modification, while also enabling remote monitoring by clinicians. However, implementation in routine practice remains limited by cost, lack of interoperability, data privacy concerns, and unequal access to technology.

### 3.7. Overall Strength of Evidence

Overall, the current literature supports personalized nutrition as a promising strategy for improving prevention and management of obesity-related metabolic disorders. A summary of the current evidence for personalized nutrition approaches across obesity, normal-weight obesity, metabolic syndrome, and MASLD is presented in Table 2 and Table 3. The strongest evidence exists for metabolic phenotyping and microbiome-informed dietary approaches, particularly in predicting postprandial glycemic responses. Moderate evidence supports the use of body composition assessment and selected nutrigenetic applications. Evidence in MASLD remains comparatively limited and requires further high-quality intervention studies. Despite rapid progress in multi-omics integration and digital health technologies, robust, large-scale randomized controlled trials are still needed to confirm clinical effectiveness, cost-effectiveness, and feasibility of implementation in routine clinical practice.

To provide an integrated overview of the maturity of personalized nutrition approaches, the available evidence was evaluated according to the overall strength of evidence, clinical readiness, major implementation barriers, and future research priorities. This summary is presented in Table 4.

## 4. Discussion

This narrative review synthesizes current evidence regarding personalized nutrition in obesity, normal-weight obesity (NWO), metabolic syndrome (MetS), and metabolic dysfunction-associated steatotic liver disease (MASLD), focusing on five complementary domains: nutrigenetics, gut microbiota, metabolic phenotyping, body composition assessment, and emerging digital health technologies. Collectively, these disorders represent a continuum of metabolic dysfunction characterized by substantial inter-individual variability in disease susceptibility, clinical presentation, progression, and response to dietary interventions [19,20,21,22,23]. This heterogeneity challenges the effectiveness of conventional “one-size-fits-all” dietary recommendations and supports the transition toward precision nutrition strategies that integrate biological, clinical, phenotypic, and behavioral characteristics. One of the principal observations emerging from this review is the limited ability of BMI alone to accurately characterize metabolic health. Although BMI remains the most widely used indicator for defining obesity, it does not distinguish between fat and lean mass or adequately reflect body fat distribution [4,5,6,9]. Consequently, individuals with similar BMI values may present markedly different metabolic phenotypes, ranging from metabolically healthy obesity to severe insulin resistance, MASLD, or cardiovascular complications. Likewise, individuals with NWO frequently remain undetected despite excess adiposity, visceral fat accumulation, chronic low-grade inflammation, and increased cardiometabolic risk [4,6,10,11,12,13,14,15,16,17,18]. These findings support growing evidence that BMI should be complemented by waist circumference, waist-to-hip ratio, waist-to-height ratio, and comprehensive body composition assessment to improve cardiometabolic risk stratification. The reviewed evidence highlights five major components that collectively shape personalized nutrition strategies.

First, nutrigenetics provides important insights into individual variability in appetite regulation, lipid metabolism, insulin sensitivity, and dietary responsiveness [19,20,21,22,23]. Nevertheless, despite increasing scientific interest, the predictive performance of currently identified genetic variants remains relatively modest. Most reported gene–diet associations explain only a limited proportion of the variability in dietary response, and many findings have not been consistently replicated across different ethnic populations. Furthermore, obesity and MASLD are highly polygenic disorders influenced by complex interactions among genetic, environmental, behavioral, and epigenetic factors. Consequently, genetic information alone is currently insufficient to guide individualized dietary recommendations in routine clinical practice. Instead, available evidence suggests that nutrigenetic information is likely to provide the greatest clinical value when integrated with metabolic phenotyping, body composition assessment, and lifestyle characteristics rather than being used as a standalone tool.

Second, the gut microbiota has emerged as an important determinant of metabolic health and dietary response. Landmark studies, including those by Zeevi et al. and the PREDICT consortium, demonstrated substantial inter-individual variability in postprandial glucose and lipid responses associated with differences in microbial composition [19,20,21,22,23]. Alterations in microbial diversity, intestinal permeability, microbial metabolites, and gut–liver axis signaling have also been implicated in obesity, insulin resistance, and MASLD progression [21,24,26,28,29]. However, despite considerable scientific interest, microbiome-based nutritional interventions remain difficult to implement in routine clinical practice. Differences in sample collection, sequencing methodologies, bioinformatic pipelines, and taxonomic classification substantially affect reproducibility across studies. Moreover, currently available predictive models have undergone only limited external validation, and relatively few large pragmatic randomized trials have demonstrated sustained clinical benefits. Consequently, although microbiome-guided dietary interventions appear promising, their clinical application requires further methodological standardization and validation.

Third, metabolic phenotyping has emerged as one of the most promising components of precision nutrition. Assessment of insulin sensitivity, glucose metabolism, circulating metabolites, inflammatory biomarkers, and energy expenditure enables stratification of individuals according to metabolic characteristics rather than BMI alone [20,21,22,23]. Such approaches may improve prediction of dietary responsiveness and facilitate individualized nutritional strategies. Nevertheless, considerable heterogeneity exists regarding biomarker selection, analytical platforms, metabolomic methodologies, and outcome measures, limiting reproducibility between studies. Standardized protocols and external validation will therefore be essential before metabolic phenotyping can be routinely incorporated into clinical decision-making.

Fourth, body composition assessment provides clinically relevant information extending beyond conventional anthropometric indices. Increasing evidence indicates that regional fat distribution, particularly visceral and ectopic adiposity, is more strongly associated with insulin resistance, hepatic steatosis, and cardiovascular risk than total body weight [4,5,6,10,11,12,13,14,15,16,17,18]. Techniques such as dual-energy X-ray absorptiometry (DXA), bioelectrical impedance analysis (BIA), magnetic resonance imaging, and computed tomography allow for more accurate characterization of adipose tissue distribution and lean body mass, thereby improving metabolic risk stratification. Nevertheless, widespread implementation remains limited by equipment availability, cost, radiation exposure for selected techniques, and the absence of universally accepted diagnostic thresholds.

Fifth, emerging digital health technologies, including wearable devices, continuous glucose monitoring, mobile health applications, artificial intelligence (AI), and machine learning algorithms, offer new opportunities for implementing personalized nutrition in clinical practice. These technologies facilitate continuous monitoring of dietary intake, physical activity, sleep, and metabolic responses while supporting individualized dietary recommendations and long-term patient engagement. However, important challenges remain, including limited external validation of predictive algorithms, insufficient interoperability between healthcare systems, implementation costs, regulatory issues, and concerns regarding data privacy and algorithm transparency. Consequently, although digital health technologies have considerable potential, their routine clinical application requires further validation in large, diverse populations. The clinical implications of these findings extend beyond obesity alone. Obesity, MetS, and MASLD share common pathogenic mechanisms, including visceral adiposity, insulin resistance, chronic inflammation, and metabolic dysregulation [2,3]. Nevertheless, considerable inter-individual variability indicates that patients with similar anthropometric characteristics may respond differently to identical dietary interventions. Personalized nutrition therefore offers an opportunity to tailor dietary recommendations according to metabolic phenotype, body composition, genetic susceptibility, gut microbiota composition, and behavioral characteristics, potentially improving treatment effectiveness and long-term adherence. Importantly, the strength of evidence differs across disease entities. Most available studies have been conducted in populations with obesity or metabolic syndrome, whereas evidence specifically addressing personalized nutrition in patients with MASLD remains comparatively limited. Although lifestyle intervention and weight reduction remain the cornerstone of MASLD management according to current international guidelines [24,26,28], relatively few randomized controlled trials have evaluated precision nutrition strategies exclusively in patients with MASLD. Consequently, many current recommendations are extrapolated from broader obesity research and should be interpreted with appropriate caution until disease-specific evidence becomes available. Our previous investigations further support the importance of comprehensive body composition assessment in pediatric MASLD. Children with MASLD exhibited significantly greater total fat mass, trunk fat mass, android fat distribution, and android-to-gynoid fat ratio than obese controls despite comparable BMI z-scores and waist circumference. These findings suggest that conventional anthropometric measures alone may underestimate clinically relevant differences in adiposity distribution. Furthermore, DXA demonstrated greater sensitivity than BIA for identifying obesity phenotypes associated with hepatic steatosis. Nevertheless, these observations originate from a pediatric cohort and therefore cannot be directly extrapolated to adult populations, highlighting the need for additional validation across different age groups. Despite encouraging progress, several important challenges continue to limit the clinical implementation of personalized nutrition. Considerable methodological heterogeneity exists among published studies regarding dietary interventions, phenotyping methods, microbiome analyses, metabolomic platforms, and outcome definitions. In addition, many proposed biomarkers require further validation, and evidence regarding long-term clinical effectiveness and cost-effectiveness remains limited. These factors currently hinder direct comparison among studies and limit translation into routine clinical practice. This review also has several limitations. Although a structured search strategy was applied, the review was conducted as a narrative synthesis rather than a systematic review or meta-analysis. Publications with different methodological designs—including randomized controlled trials, observational studies, mechanistic investigations, systematic reviews, and consensus statements—were synthesized qualitatively, and no formal risk-of-bias assessment was performed. Consequently, the conclusions should be interpreted as an integrated overview of current evidence rather than a quantitative evaluation of intervention effectiveness. Moreover, only English-language publications were included, introducing the possibility of publication and language bias. Future progress in personalized nutrition will depend on the integration of genetic, metabolic, phenotypic, behavioral, environmental, and digital health information into clinically applicable decision-support systems, consistent with the conceptual framework proposed by Ordovás et al. [27,30]. Future research should prioritize standardized metabolic phenotyping protocols, harmonization of microbiome analyses, validation of clinically useful biomarkers, cost-effectiveness analyses, and adequately powered randomized controlled trials with long-term follow-up, particularly in patients with MASLD. Close collaboration among clinicians, dietitians, molecular biologists, bioinformaticians, and digital health specialists will be essential to facilitate the responsible translation of precision nutrition into routine clinical practice.

## 5. Conclusions

Personalized nutrition represents a promising but still evolving approach to the prevention and management of obesity, normal-weight obesity (NWO), metabolic syndrome (MetS), and metabolic dysfunction-associated steatotic liver disease (MASLD). By integrating body composition assessment, metabolic phenotyping, genetic susceptibility, gut microbiota profiling, and digital health technologies, precision nutrition has the potential to improve risk stratification and support more individualized dietary interventions beyond conventional BMI-based approaches. However, the current evidence remains heterogeneous, and several important challenges continue to limit routine clinical implementation. The predictive value of many genetic and microbiome-based biomarkers remains modest, standardized analytical methodologies are lacking, and evidence from large, long-term randomized controlled trials—particularly in patients with MASLD—is still limited. Consequently, personalized nutrition should currently be regarded as a complementary strategy rather than a replacement for established evidence-based lifestyle interventions. Future research should focus on validating clinically useful biomarkers, harmonizing phenotyping and multi-omics methodologies, evaluating cost-effectiveness, and conducting adequately powered randomized controlled trials to determine which personalized nutrition strategies provide meaningful clinical benefits for specific patient populations. Addressing these challenges will be essential for translating precision nutrition into safe, effective, and sustainable routine clinical practice.

## 6. Strengths and Limitations

This review has several limitations. First, it was conducted as a narrative review rather than a systematic review or meta-analysis, and therefore the selection and interpretation of the available evidence may be subject to a greater degree of subjective judgment. Second, the included publications were highly heterogeneous with respect to study design, study populations, dietary interventions, outcome measures, and analytical methodologies, precluding quantitative synthesis and limiting direct comparisons across studies. Third, evidence from randomized controlled trials, observational studies, mechanistic investigations, systematic reviews, and consensus documents was synthesized qualitatively; consequently, the strength of evidence differs substantially across the thematic domains discussed. Fourth, relatively few studies have specifically investigated personalized nutrition in patients with MASLD, and several conclusions are therefore extrapolated from broader populations with obesity or metabolic syndrome. In addition, only English-language publications were included, introducing the possibility of publication and language bias. Finally, because precision nutrition, multi-omics technologies, and digital health are rapidly evolving fields, future high-quality evidence may further refine the conclusions presented in this review.

## Figures and Tables

**Table 1:** Representative studies investigating personalized nutrition approaches in obesity, metabolic syndrome, normal-weight obesity, and metabolic dysfunction-associated steatotic liver disease (MASLD).

Study (Year)	Study Design	Population	Personalization Strategy	Main Findings/Clinical Relevance
A. Randomized controlled and intervention studies				
Zeevi et al. (2015) [19]	Controlled intervention study	Healthy adults	Personalized prediction of postprandial glucose responses using clinical and microbiome data	Demonstrated substantial inter-individual variability in glycemic responses and the potential of microbiome-informed dietary recommendations
Personalized dietary intervention studies [25]	RCT	Individuals with metabolic risk	Individualized dietary recommendations based on metabolic phenotype and/or lifestyle characteristics	Reported improvements in selected metabolic outcomes; however, clinical effects remain heterogeneous
B. Cohort and mechanistic precision nutrition studies				
PREDICT 1 (Berry et al., 2020) [20]	Prospective cohort study	>1000 healthy and at-risk adults	Integration of metabolic, dietary, genetic, and microbiome data	Identified marked variability in postprandial metabolic responses and predictors of individual dietary responses
PREDICT 2 (2021) [21]	Twin and cohort study	Adult twins and unrelated individuals	Multi-omics integration and metabolic phenotyping	Demonstrated the contribution of genetic and non-genetic factors to variability in metabolic responses
Berry et al. (2020) [23]	Precision nutrition study	Healthy adults	Multi-omics profiling and metabolic characterization	Identified biological predictors associated with differential responses to dietary exposure
Qi et al. (2018) [26]	Gene–diet interaction study	Adults with obesity	FTO genotype-based dietary response assessment	Suggested genotype-dependent differences in weight-loss response; clinical applicability remains uncertain
C. Evidence related to MASLD and metabolic complications				
Rinella et al. (2023) [24]	Clinical practice guidance/review	Patients with MASLD	Precision nutrition framework	Discussed the potential role of individualized dietary strategies in MASLD management and highlighted current evidence limitations
Mediterranean diet intervention studies in MASLD [27]	RCT/cohort studies	Patients with MASLD	Dietary pattern personalization based on metabolic profile and clinical characteristics	Reported reductions in hepatic steatosis and improvements in metabolic parameters; long-term personalized effects require further validation

Note: Studies initially referring to non-alcoholic fatty liver disease (NAFLD) are described using the updated terminology metabolic dysfunction-associated steatotic liver disease (MASLD), according to current nomenclature. Different study designs provide different levels of evidence; randomized controlled trials were considered most informative for evaluating clinical effectiveness, whereas cohort and mechanistic studies primarily contributed evidence regarding associations, biological mechanisms, and potential predictors of dietary response.

**Table 2:** Clinical evidence supporting personalized nutrition in obesity, metabolic syndrome, and MASLD.

Study	Population	Disease	Personalized Nutrition Approach	Main Outcome	Clinical Significance
Zeevi et al. (2015) [19]	800 healthy adults	Obesity risk	Machine learning + gut microbiome	Accurate prediction of postprandial glycemic responses	First proof-of-concept for precision nutrition
Food4Me Consortium (2015)	1607 adults	Overweight/obesity	Personalized dietary advice based on phenotype, diet, and genotype	Improved dietary adherence	Demonstrated feasibility of personalized nutrition in Europe
Berry et al. (2020) [20] (PREDICT 1)	>1000 adults	Obesity/MetS	Multi-omics + metabolic phenotyping	Large inter-individual metabolic variability	Established biological basis for individualized nutrition
Asnicar et al. (2021) [21] (PREDICT 2)	Twins and unrelated adults	Obesity/MetS	Gut microbiome + metabolomics	Improved prediction of metabolic responses	Confirmed reproducibility of precision nutrition algorithms
Ben-Yacov et al. (2021) [22]	Adults with prediabetes	MetS	Personalized postprandial diet	Better glycemic control than Mediterranean diet	Clinical validation of personalized nutrition
Qi et al. [26]	Adults with obesity	Obesity	Nutrigenetics (*FTO*)	Genotype influenced weight-loss response	Supports gene–diet interaction
Romero-Corral et al. [4]	Adults	NWO	Body composition assessment	Increased cardiometabolic risk despite normal BMI	Introduced NWO phenotype
Oliveros et al. [6]	Review	NWO	Phenotypic assessment	Confirmed limitations of BMI	Supports body composition assessment
Recent MASLD intervention study (2024–2026)	Adults with MASLD	MASLD	Mediterranean diet + personalized counseling	Reduced liver fat	Emerging clinical evidence
Recent MASLD intervention study (2025–2026)	Adults with MASLD	MASLD	Low-carbohydrate personalized diet	Improved insulin resistance	Supports individualized treatment

**Table 3:** Evidence synthesis according to the five pillars of personalized nutrition.

Personalized Nutrition Domain	Biological Basis	Main Clinical Applications	Evidence in Obesity	Evidence in MetS	Evidence in MASLD	Current Limitations
Nutrigenetics	Gene–diet interactions	Individualized dietary recommendations	Moderate	Moderate	Limited	Small effect sizes, inconsistent replication
Gut microbiota	Gut microbial diversity and metabolites	Prediction of glycemic responses	Strong	Strong	Moderate	Lack of standardized analytical methods
Metabolic phenotyping	Metabolomics, insulin sensitivity	Stratification of dietary responders	Strong	Strong	Moderate	High analytical complexity
Body composition assessment	DXA, BIA, MRI, visceral fat	Beyond BMI risk assessment	Strong	Strong	Strong	Limited access to advanced imaging
Digital health technologies	AI, CGM, wearables, mobile applications	Continuous monitoring and personalized feedback	Moderate	Moderate	Emerging	Cost, interoperability, data privacy

**Table 4:** Clinical readiness, strength of evidence, and future priorities.

Personalized Nutrition Domain	Number of Clinical Studies	Overall Strength of Evidence	Clinical Readiness	Main Barriers	Research Priorities
Nutrigenetics	High	Moderate	Moderate	Limited predictive value of single polymorphisms	Polygenic risk models
Gut microbiota	High	Strong	Moderate	Standardization of microbiome analysis	Large multicenter RCTs
Metabolic phenotyping	Moderate–High	Strong	High	Cost of metabolomics	Clinical implementation studies
Body composition assessment	High	Strong	High	Access to DXA/MRI in some settings	Integration into clinical algorithms
Digital health technologies	Moderate	Moderate	Moderate	Cost, reimbursement, interoperability	AI-assisted decision support
Multi-omics integration	Emerging	Limited–Moderate	Low	High cost, data integration, lack of standardization	Precision nutrition platforms and implementation science

## Data Availability

No new data were created or analyzed in this study. Data sharing is not applicable to this article.

## References

[1] Kelly T., Yang W., Chen C.S., Reynolds K., He J. (2008). Global burden of obesity in 2005 and projections to 2030. Int. J. Obes..

[2] Apovian C.M. (2016). Obesity: Definition, comorbidities, causes, and burden. Am. J. Manag. Care.

[3] Tremmel M., Gerdtham U.G., Nilsson P.M., Saha S. (2017). Economic burden of obesity: A systematic literature review. Int. J. Environ. Res. Public Health.

[4] Romero-Corral A., Somers V.K., Sierra-Johnson J., Korenfeld Y., Boarin S., Korinek J., Jensen M.D., Parati G., Lopez-Jimenez F. (2010). Normal weight obesity: A risk factor for cardiometabolic dysregulation and cardiovascular mortality. Eur. Heart J..

[5] Rothman K.J. (2008). BMI-related errors in the measurement of obesity. Int. J. Obes..

[6] Oliveros E., Somers V.K., Sochor O., Goel K., Lopez-Jimenez F. (2014). The concept of normal weight obesity. Prog. Cardiovasc. Dis..

[7] Finkelstein E.A., Khavjou O.A., Thompson H., Trogdon J.G., Pan L., Sherry B., Dietz W. (2012). Obesity and severe obesity forecasts through 2030. Am. J. Prev. Med..

[8] Luhar S., Timæus I.M., Jones R., Cunningham S., Patel S.A., Kinra S., Clarke L., Houben R. (2020). Forecasting the prevalence of overweight and obesity in India to 2040. PLoS ONE.

[9] Robinson E. (2017). Overweight but unseen: A review of the underestimation of weight status. Obes. Rev..

[10] Bellissimo M.P., Cai Q., Ziegler T.R., Liu K.H., Tran P.H., Vos M.B., Martin G.S., Jones D.P., Yu T., Alvarez J.A. (2019). Plasma high-resolution metabolomics differentiates adults with normal weight obesity from lean individuals. Obesity.

[11] Berg C., Strandhagen E., Mehlig K., Subramoney S., Lissner L., Björck L. (2015). Normal weight adiposity in a Swedish population. Obes. Sci. Pract..

[12] Cho W.K., Kim H., Lee H.Y., Han K.D., Jeon Y.J., Jung I.A., Kim S.H., Cho K.S., Park S.H., Jung M.H. (2015). Insulin resistance of normal weight central obese adolescents in Korea. Int. J. Endocrinol..

[13] Kim M.K., Han K., Kwon H.S., Song K., Yim H.W., Lee W., Park Y. (2014). Normal weight obesity in Korean adults. Clin. Endocrinol..

[14] Jia A., Xu S., Xing Y., Zhang W., Yu X., Zhao Y., Ming J., Ji Q. (2018). Prevalence and cardiometabolic risks of normal weight obesity in Chinese population. Nutr. Metab. Cardiovasc. Dis..

[15] Madeira F.B., Silva A.A., Veloso H.F., Goldani M.Z., Kac G., Cardoso V.C., Bettiol H., Barbieri M.A. (2013). Normal weight obesity is associated with metabolic syndrome and insulin resistance in young adults from a middle-income country. PLoS ONE.

[16] Jean N., Somers V.K., Sochor O., Medina-Inojosa J., Llano E.M., Lopez-Jimenez F. (2014). Normal-weight obesity and cardiovascular health. Curr. Atheroscler. Rep..

[17] Suliga E., Kozieł D., Głuszek S. (2016). Metabolic syndrome in normal weight individuals. Ann. Agric. Environ. Med..

[18] Conus F., Rabasa-Lhoret R., Peronnet F. (2007). Metabolically obese normal-weight subjects. Appl. Physiol. Nutr. Metab..

[19] Zeevi D., Korem T., Zmora N., Israeli D., Rothschild D., Weinberger A., Ben-Yacov O., Lador D., Avnit-Sagi T., Lotan-Pompan M. (2015). Personalized nutrition by prediction of glycemic responses. Cell.

[20] Berry S.E., Valdes A.M., Drew D.A., Asnicar F., Mazidi M., Wolf J., Capdevila J., Hadjigeorgiou G., Davies R., Al Khatib H. (2020). Human postprandial responses to food and potential for precision nutrition. Nat. Med..

[21] Asnicar F., Berry S.E., Valdes A.M., Nguyen L.H., Piccinno G., Drew D.A., Leeming E., Gibson R., Le Roy C., Khatib H.A. (2021). Microbiome and metabolic phenotyping Microbiome connections with host metabolism and habitual diet from 1098 deeply phenotyped individuals. Nat. Med..

[22] Ben-Yacov O., Godneva A., Rein M., Shilo S., Kolobkov D., Koren N., Dolev N.C., Shmul T.T., Wolf B.C., Kosower N. (2021). Personalized Postprandial Glucose Response–Targeting Diet Versus Mediterranean Diet for Glycemic Control in Prediabetes. Diabetes Care.

[23] Berry S.E., Tydeman E.A., Lewis H.B., Phalora R., Rosborough J., Picout D.R., Ellis P.R. (2008). Manipulation of lipid bioaccessibility of almond seeds influences postprandial lipemia in healthy human subjects. Am. J. Clin. Nutr..

[24] Rinella M.E., Lazarus J.V., Ratziu V., Francque S.M., Sanyal A.J., Kanwal F., Romero D., Abdelmalek M.F., Anstee Q.M., Arab J.P. (2023). A multi-society Delphi consensus statement on new fatty liver disease nomenclature. J. Hepatol..

[25] Francque S.M., Marchesini G., Kautz A., Walmsley M., Dorner R., Lazarus J.V., Zelber-Sagi S., Hallsworth K., Busetto L., Frühbeck G. (2021). Non-alcoholic fatty liver disease: A patient guideline. JHEP Rep..

[26] Qi L. (2022). Nutrition for precision health: The time is now. Obesity.

[27] Seral-Cortes M., Drouard G., Masip G., Bogl L.H., De Henauw S., Foraita R., Intemann T., Lissner L., Molnar D., Nagrani R. (2025). Mediterranean diet and obesity polygenic risk interaction on adiposity in European children: The IDEFICS/I.Family Study. Pediatr. Obes..

[28] European Association for the Study of the Liver (EASL), European Association for the Study of Diabetes (EASD), European Association for the Study of Obesity (EASO) (2024). EASL–EASD–EASO Clinical Practice Guidelines on the management of metabolic dysfunction-associated steatotic liver disease (MASLD). J. Hepatol..

[29] Kanwal F., Bril F., Wong V.W.-S., Adams L.A., Pfotenhauer K., Skolnik N., Wright E.E., Eckel R.H., Noureddin M., Loomba R. (2026). Clinical Care Pathway for the Risk Stratification and Management of Patients with Metabolic Dysfunction-Associated Steatotic Liver Disease. Gastroenterology.

[30] Ordovas J.M., Ferguson L.R., Tai E.S., Mathers J.C. (2018). Personalised nutrition and health. BMJ.

